# Intergenerational Metabolomic Analysis of Mothers with a History of Gestational Diabetes Mellitus and Their Offspring

**DOI:** 10.3390/ijms21249647

**Published:** 2020-12-17

**Authors:** Raffael Ott, Xenia Pawlow, Andreas Weiß, Anna Hofelich, Melanie Herbst, Nadine Hummel, Cornelia Prehn, Jerzy Adamski, Werner Römisch-Margl, Gabi Kastenmüller, Anette-G. Ziegler, Sandra Hummel

**Affiliations:** 1 Institute of Diabetes Research, Helmholtz Zentrum München, and Forschergruppe Diabetes, Klinikum rechts der Isar, Technische Universität München, 85764 Neuherberg, Germany; Raffael.ott@helmholtz-muenchen.de (R.O.); xenia.pawlow@gmx.de (X.P.); andreas.weiss@helmholtz-muenchen.de (A.W.); anna.hofelich@helmholtz-muenchen.de (A.H.); Melanie.Herbst@helmholtz-muenchen.de (M.H.); nadine-hummel@gmx.de (N.H.); anette-g.ziegler@helmholtz-muenchen.de (A.-G.Z.); 2 Forschergruppe Diabetes e.V., 85764 Neuherberg, Germany; 3 Research Unit Molecular Endocrinology and Metabolism, Genome Analysis Center, Helmholtz Zentrum München, 85764 Neuherberg, Germany; prehn@helmholtz-muenchen.de (C.P.); adamski@helmholtz-muenchen.de (J.A.); 4 Chair for Experimental Genetics, Technical University of Munich, 85354 Freising-Weihenstephan, Germany; 5 Department of Biochemistry, Yong Loo Lin School of Medicine, National University of Singapore, Singapore 117597, Singapore; 6 German Center for Diabetes Research (DZD), München-Neuherberg, 85764 Neuherberg, Germany; werner.roemisch@helmholtz-muenchen.de (W.R.-M.); g.kastenmueller@helmholtz-muenchen.de (G.K.); 7 Institute of Computational Biology, Helmholtz Zentrum München, 85764 Neuherberg, Germany

**Keywords:** gestational diabetes, overweight, intergenerational metabolomics, lifestyle

## Abstract

Shared metabolomic patterns at delivery have been suggested to underlie the mother-to-child transmission of adverse metabolic health. This study aimed to investigate whether mothers with gestational diabetes mellitus (GDM) and their offspring show similar metabolomic patterns several years postpartum. Targeted metabolomics (including 137 metabolites) was performed in plasma samples obtained during an oral glucose tolerance test from 48 mothers with GDM and their offspring at a cross-sectional study visit 8 years after delivery. Partial Pearson’s correlations between the area under the curve (AUC) of maternal and offspring metabolites were calculated, yielding so-called Gaussian graphical models. Spearman’s correlations were applied to investigate correlations of body mass index (BMI), Matsuda insulin sensitivity index (ISI-M), dietary intake, and physical activity between generations, and correlations of metabolite AUCs with lifestyle variables. This study revealed that BMI, ISI-M, and the AUC of six metabolites (carnitine, taurine, proline, SM(-OH) C14:1, creatinine, and PC ae C34:3) were significantly correlated between mothers and offspring several years postpartum. Intergenerational metabolite correlations were independent of shared BMI, ISI-M, age, sex, and all other metabolites. Furthermore, creatinine was correlated with physical activity in mothers. This study suggests that there is long-term metabolic programming in the offspring of mothers with GDM and informs us about targets that could be addressed by future intervention studies.

## 1. Introduction

The prevalence of overweight and obesity is increasing worldwide, and rates of increase have been reported to be the highest in early adulthood, including women entering pregnancy [1]. In parallel, a rise of pregnancies complicated by gestational diabetes mellitus (GDM) has been observed [2]. There is compelling evidence that exposure to a maternal obese and, in particular, diabetic environment in utero influences the offspring’s risk for metabolic disorders over the life course. It has been suggested that genetic susceptibility and an adverse intrauterine environment contribute independently to the early programming of childhood overweight and adverse metabolic outcomes [3,4], although the underlying mechanisms are not yet fully understood. Another hypothesis is that the transmission of overweight risk is attributable to lifestyle behaviors shared between mothers and their offspring. However, only a few studies have investigated whether mothers and children share similar lifestyle behaviors. While one Chinese study reported similarities in some dietary and activity behaviors between children and their parents [5], fewer associations were observed in a Swedish study [6].

Plasma metabolite levels provide an objective read-out of the individual biological status arising from genetic and environmental interactions and have been widely used in studies with the aim to identify either biomarkers or pathways underlying chronic diseases, including obesity and diabetes [7,8]. Previous reports suggested a correlation of maternal metabolites during the peripartum period and cord blood metabolites, which led to the hypothesis that metabolomic patterns are shared between mothers and their offspring during this early stage of life, and that these may be disease relevant [9,10]. However, it remained unclear from these studies whether shared metabolomic patterns could still be observed several years postpartum. The results from a population-based cohort reported only weak correlations between plasma metabolites of healthy pregnant women and their offspring at the age of 10 years [11]. This study was limited to metabolomics analysis in randomly collected non-fasting samples. The assessment of plasma metabolites in response to standardized glucose challenge has been shown to improve the detection of metabolic alterations [12,13], therefore, this method may be better suited to detect intergenerational metabolomic correlations in the context of obesity and diabetes. Furthermore, in line with the hypothesis that transmission of the risk of disturbed metabolism from one generation to the next is related to the transmission of metabolic pathways, it would be reasonable that similarities in metabolic pathways are more likely to occur in the high-risk group of mothers with GDM and their children than in a population-based cohort.

We therefore used a targeted metabolomics approach in a cohort of mothers with GDM and their offspring who participated in a clinical study visit 8 years after delivery with the aim to investigate: (1) whether plasma metabolite levels in response to glucose challenge are correlated between mothers and offspring several years postpartum independent of postpartum BMI and insulin sensitivity, which may also be shared between mothers and their offspring, and (2) whether intergenerational metabolomics may be affected by dietary glycemic load or physical activity behavior.

## 2. Results

### 2.1. Characteristics of the Cohort

Targeted metabolomics analysis was performed in 288 plasma samples collected from 48 mothers with GDM and their offspring during an oral glucose tolerance test (OGTT) at a postpartum study visit at a median of 8 years after delivery (interquartile range (IQR) 5.6; 8.8 years; Table 1).

At the postpartum study visit, mothers had a median body mass index (BMI) of 26.4 kg/m^2^ (IQR 22.6; 32 kg/m^2^), including 28 (58%) mothers with overweight, and their offspring had a median BMI-standard deviation score (SDS) of 0.1 (IQR −0.6; 0.8), including 7 (15%) offspring with overweight. At the same visit, mothers had a median Matsuda insulin sensitivity index (ISI-M) of 6.1 (IQR 3.7; 8.8) and their offspring had a median ISI-M of 10.3 (IQR 6.6; 13.7). BMI was inversely correlated with ISI-M in mothers (r = −0.69, *p* < 0.0001; Table 2) and in offspring (r = −0.32, *p* < 0.05). At the postpartum clinical study visit, 17 of 48 mothers had either impaired fasting glucose or impaired glucose tolerance. Information on dietary glycemic load was available for 47 mothers and 31 offspring and information on physical activity behavior (metabolic equivalent of task hours (MET-h)/week) was available for 37 mothers and 20 offspring (Table 1). Within generations, dietary glycemic load was correlated with physical activity (MET-h/week) in offspring of mothers with GDM (r = 0.51, *p* < 0.05). No significant correlations were observed between dietary glycemic load or physical activity and BMI/BMI-SDS and ISI-M within generations (Table 2).

Compared to the offspring included in this study, offspring who were excluded due to missing OGTT samples were younger at the clinical study visit (median age 5.1 years (IQR 4.1; 7.4) vs. 8 years (5.6; 8.8), *p* < 0.0001). Included mother–offspring pairs did not significantly differ from excluded pairs with respect to country of origin, offspring sex, maternal and offspring BMI/BMI-SDS, and fasting glucose (Appendix A).

### 2.2. Intergenerational Correlations of BMI, Insulin Sensitivity, and Lifestyle

**Figure 1:**
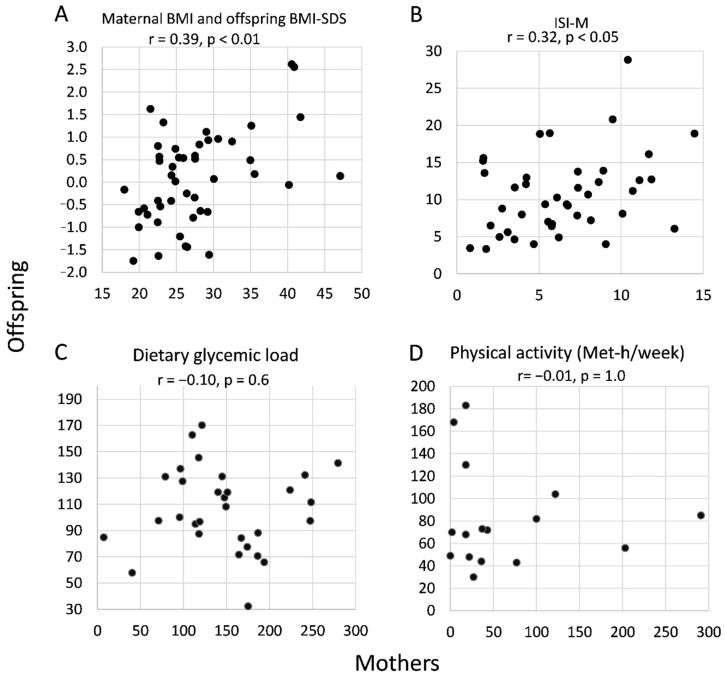
Correlation plots of (**A**) maternal BMI and offspring BMI-SDS, (**B**) ISI-Matsuda, (**C**) dietary glycemic load, and (**D**) physical activity (MET-h/week) between mothers with GDM and their offspring assessed at a median of 8 years after delivery. Correlations were assessed by Spearman’s correlation test.

Between generations, significant correlations were observed between maternal BMI and offspring BMI-SDS (r = 0.39, *p* < 0.01; Figure 1A) and between maternal and offspring ISI-M (r = 0.32, *p* < 0.05; Figure 1B). No significant correlations were observed between maternal and offspring dietary glycemic load or physical activity behavior (Figure 1C,D).

Similarly, no significant correlations were observed between maternal and offspring intake of energy, protein, carbohydrates, fiber, total fat, and poly- and mono-unsaturated and saturated fatty acids and maternal and offspring Dietary Approaches to Stop Hypertension (DASH) score (Appendix B).

### 2.3. Intergenerational Correlations of AUC of Metabolites

**Figure 2:**
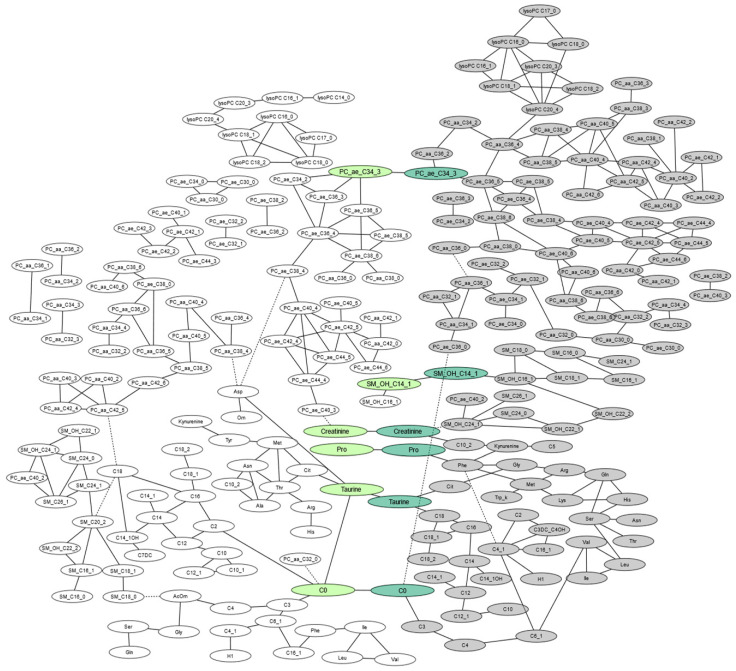
Intra- and intergenerational correlations of area under the curve (AUC) of plasma metabolite levels assessed at 0, 30, and 120 min during a 75 g oral glucose tolerance test (OGTT) between mothers with gestational diabetes mellitus (GDM) (open ovals) and their offspring (filled ovals) at a median of 8 years after delivery. Each node represents the AUC of a plasma metabolite and edges between two nodes represent significant partial correlation of two metabolites at a false discovery rate (FDR) corrected *p*-value < 0.05, adjusted for age, sex, BMI/BMI-SDS, and ISI-M, and for the remaining metabolites. Intergenerationally correlated metabolites are highlighted in light green for maternal and dark green for offspring. Network figure is restricted to intragenerational correlations at r > 0.06 (the smallest correlation coefficient of intergenerational correlations at FDR *p* < 0.05).

**Figure 3:**
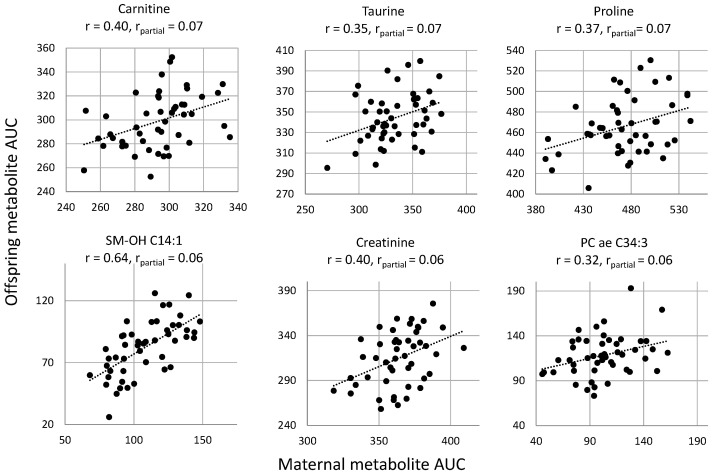
Correlation plots of AUC of carnitine, taurine, proline, SM –(OH) C14:1, creatinine, and PC ae C34:3 between mothers with GDM and their offspring assessed at a median of 8 years after delivery. Metabolites were identified to be significantly correlated between mothers and offspring based on partial Pearson’s correlations (GGM), at FDR corrected *p*-value < 0.05. Correlation estimates are shown for both Pearson’s and partial Pearson’s correlations derived from GGM.

Gaussian graphical model (GGM) analysis including 137 maternal and 137 offspring plasma metabolites revealed that the AUC of 6 metabolites in response to glucose challenge was significantly correlated between mothers and offspring several years after delivery, after adjusting for maternal and offspring BMI/BMI-SDS, ISI-M, age, offspring sex, and all other metabolites and after correction for multiple testing (Figure 2, Appendix C). These included intergenerational correlations of the AUC of free carnitine (C0, r_partial_ = 0.07, false discovery rate (FDR) *p* < 0.01), one glycerophospholipid (PC ae C34:3 r_partial_ = 0.06, FDR *p* < 0.05), two biogenic amines (taurine, r_partial_ = 0.07, FDR *p* < 0.05; creatinine, r_partial_ = 0.06, FDR *p* = 0.01), one amino acid (proline, r_partial_ = 0.07, FDR *p* < 0.01), and one sphingolipid (SM -(OH) C14:1, r_partial_ = 0.06, FDR *p* < 0.01). Note that, in general, correlation estimates resulting from GGM are notably lower than those resulting from regular Pearson’s or Spearman’s correlations [14]. To visualize this, maternal and offspring AUC values of the intergenerationally correlated metabolites and Pearson’s correlation coefficients compared to partial correlation coefficient are shown in Figure 3. In the mothers, the AUC values of the intergenerationally correlated metabolites were not associated with a diagnosis of either impaired fasting glucose or impaired glucose tolerance (Appendix D).

GGM analysis further showed a clear separation into maternal and offspring metabolite clusters. Notably, within these two clusters, the network showed modular structures with respect to the metabolite classes in our panel, such as lyso-phosphatidylcholines (Lyso-PCs), branched chain amino acids (BCAAs), and acylcarnitines, with similar modules in both generations, capturing known biochemical pathways. Metabolites, fasting levels of which had been strongly correlated in previously published GGM-derived networks and which are known to be in close proximity in the metabolic network [14], were significantly correlated within generations in response to glucose challenge in this study, such as Lyso-PC a C16:0 with Lyso-PC a C18:0 (r_partial_ = 0.118, Pearson correlation r = 0.922, FDR *p* < 0.0001) and leucine with valine (r_partial_ = 0.115, Pearson correlation r = 0.88, FDR *p* < 0.0001) in the offspring, and methionine and threonine (r_partial_ = 0.11, Pearson correlation 0.7, FDR *p* < 0.0001) in the mothers (Figure 2, Appendix C).

When performing separate GGM analysis on maternal and offspring metabolite levels at 30 and 120 min after glucose challenge, we observed significant intergenerational correlations of taurine (r_partial_ = 0.08, FDR *p* < 0.01) and C0 (r_partial_ = 0.07, FDR *p* < 0.05) at 30 min, and significant intergenerational correlations of proline (r_partial_ = 0.07, FDR *p* < 0.01) at 120 min.

### 2.4. Effect of Lifestyle on Intergenerationally Correlated Metabolites

In the next step, we investigated within generations whether postpartum dietary glycemic load or physical activity was correlated with the AUC of intergenerationally correlated plasma metabolites. In mothers, physical activity was correlated with the AUC of creatinine levels, (r = 0.50, *p* < 0.01; Table 3). No correlations between maternal dietary glycemic load and the AUC of metabolites were observed.

In offspring, intergenerationally correlated metabolites were not significantly correlated with physical activity or dietary glycemic load (Table 3).

## 3. Discussion

This explorative study indicates that several years after delivery, a number of plasma metabolite levels, assessed as AUC in response to glucose challenge, were correlated between mothers with GDM and their offspring. The intergenerationally correlated metabolites included carnitine (C0), one glycerophospholipid (PC ae C34:3), two biogenic amines (taurine, creatinine), one amino acid (proline), and one sphingolipid (SM –(OH) C14:1).

Our study further identified that BMI (BMI-SDS in the offspring) and ISI-M were correlated between mothers and offspring several years postpartum. In numerous studies, BMI and insulin sensitivity have been linked to alterations in plasma levels of several metabolites [7,15]. The metabolites identified in our study correlated between mothers and offspring after adjusting for maternal and offspring BMI/BMI-SDS and ISI-M, indicating that they were independent of shared postpartum BMI or insulin sensitivity.

Of interest, all intergenerationally correlated metabolites have been linked to the pathogenesis of obesity and/or diabetes in prior studies and may therefore be indicators of shared metabolic health in mother–offspring pairs [16,17,18,19,20,21]. Due to the cross-sectional design of our study, we were not able to assess whether the intergenerational metabolite correlations may persist from birth. However, few existing studies have shown a correlation between maternal levels during pregnancy and cord blood levels of carnitine [9,11] and proline [10,11] or between maternal levels during pregnancy and childhood levels of PC ae C34:3 [11]. It remained unclear from these studies whether the intergenerational correlations result from transplacental transfer of these metabolites or rather reflect shared metabolic pathways. Our study suggests that the intergenerational correlations of carnitine, proline, and PC ae C34:3 persist from the prenatal period to childhood, supporting the hypothesis of shared metabolic patterns. Moreover, based on our data, there is no indication that these intergenerational correlations result from shared lifestyle behavior, as dietary glycemic load, energy and macronutrient intake, modified DASH score, and physical activity were not correlated between mothers and offspring. Moreover, plasma AUCs of carnitine, proline, and PC ae C34:3 were not correlated with physical activity or dietary glycemic load within generations. This is consistent with results from a lifestyle intervention study in obese children, where proline and PC ae C34:3 plasma levels were not affected by weight loss due to changes in diet or physical activity [22]. Since all three metabolites were reported to correlate between mothers and offspring already at the time of delivery, their intergenerational correlations may be caused by factors related to pregnancy or even the time before pregnancy. In this context, the impact of shared genetic or epigenetic factors on intergenerational metabolite correlations needs to be further investigated.

While we found no associations of carnitine, proline, and PC ae C34:3 with lifestyle behavior, our study indicated that the AUC of creatinine in response to glucose challenge may be affected by physical activity, consistent with a report on increased plasma creatinine levels with increased muscle cell mass [23]. Unlike the mothers, we could not observe any correlation between physical activity and creatinine in the offspring. This could be due to the small sample size, but also to age and gender effects or differences in the type of physical activity between mothers and their children. Further studies are warranted to investigate the metabolomic response to physical activity in women and children at increased risk for obesity and type 2 diabetes.

To our knowledge, this is the first study to quantify a broad spectrum of metabolites in a cohort of mothers diagnosed with GDM using a standardized OGTT and their offspring several years postpartum. The primary strength of our study was the analysis of metabolomics in mother–offspring pairs in response to OGTT, as previous studies highlighted that the detection of inter-individual differences and disease-predictive metabolic trajectories is improved when assessing metabolomics in response to food challenges [13,24]. This is supported by findings from our study, in which the AUC of metabolites combined the dynamic changes in response to glucose challenge that were observed at separate time-points and identified additional intergenerationally correlated metabolites. Furthermore, by calculating the AUC of metabolites, we have minimized the risk of bias that may be caused by the inclusion of metabolites with concentrations below the detection limit when analyzing individual time points. In contrast to previous studies, we used GGM to study intergenerational metabolite correlations. While standard correlation-based methods lack the ability to discriminate between direct and indirect associations, GGM identifies the independence between two metabolites conditional on all others and has been suggested as an effective tool in metabolomics analysis [14]. In this data-driven network, we observed a clear separation of maternal and offspring metabolites into two separate clusters, and within these two clusters, the network showed modular structures of metabolites belonging to the same class (glycerophospholipids, with substructures of Lyso-PCs, PCaa, and PCae; sphingolipids; amino acids; acylcarnitines; biogenic amines; and hexoses).

We further observed within generations that metabolites that are known to be one reaction step apart were often strongly correlated, and thus were neighbors in the maternal–offspring network clusters. For example, the amino acids leucine and valine in the children and methionine and threonine in the mothers were among the strongest intragenerational correlations in the data-driven network and are known to share similar biosynthesis and degradation pathways. Thus, consistent with the results of previous studies on fasting metabolite concentrations [25], our GGM delivers plausible biochemical networks. In addition to intragenerational metabolite clusters, the network analysis further identified significant correlations of six metabolite AUCs between mothers and children, after regressing out correlations of these metabolites with other within- and across-generation metabolites. As the GGM resembles known biology regarding the physiological differences between generations and metabolites sharing the same metabolic pathways, we believe that the identified intergenerational correlations may indicate similarities in some of the metabolomic pathways in mothers and their offspring. Furthermore, the assessment of detailed postpartum lifestyle data enabled an explorative investigation of their potential impact on intergenerational metabolomics.

There are some limitations in our study. First, we were not able to investigate the persistence of intergenerational metabolite correlations in a longitudinally followed cohort. Therefore, the interpretation of our results with respect to persistency is based on results from previous studies that included different cohorts and needs to be confirmed by longitudinal studies. Our study was further limited by the overall small sample size, and specifically the availability of lifestyle data. It is thus possible that weaker intergenerational metabolite correlations or correlations between lifestyle variables and metabolite levels were missed.

In conclusion, our study suggests that several metabolic patterns, which may be disease relevant, are shared between mothers with GDM and their offspring several years postpartum, independent of shared BMI or insulin sensitivity. Of those, some seem to persist from the time of delivery and are not affected by lifestyle behavior, suggesting long-term programming of metabolic health in the children of mothers with gestational diabetes. These findings inform us about metabolite targets that could be addressed by future intervention studies to promote long-term metabolic health in these children.

## 4. Materials and Methods

### 4.1. Postpartum Outcomes in Women with Gestational Diabetes and Their Offspring (POGO) Study

Intergenerational metabolomic profiles were assessed in mothers diagnosed with GDM (according to the guidelines of the German Diabetes Association from 2001) during their most recent pregnancy and in their offspring, from plasma samples obtained during an oral glucose tolerance test (OGTT) performed at a postpartum visit within the observational Postpartum Outcomes in Women with Gestational Diabetes and Their Offspring (POGO) study. The POGO study was conducted between March 2011 and November 2013 in Munich, Germany. Details of the study are described elsewhere [26]. Briefly, the study recruited women who were referred for screening of GDM to an outpatient clinic during at least one pregnancy between 1998 and 2009 to participate in a clinical examination once within 3–12 years after delivery. Because screening of GDM was not a regular part of the pregnancy check-ups in Germany before 2012, women were usually screened if they were at increased risk for GDM (e.g., family history of diabetes, GDM in a previous pregnancy, previous birth of a large-for-gestational-age infant, habitual abortion, fetal macrosomia), or because glucosuria and/or hyperglycemia were detected. All the women and their children participated in a postpartum clinical examination on the same day at the clinical study center of the Institute of Diabetes Research, Helmholtz Zentrum München, Germany. During the examination, the participants, who had fasted for at least 8 h, underwent a 75 g OGTT (Dextro O.G.T.; Roche Diagnostics, Mannheim, Germany), and plasma samples for glucose and insulin assessment and metabolomics profiling were collected at 0, 30, and 120 min. The time from blood collection to centrifugation was <40 min, and for metabolomics analysis, the plasma was transferred to precooled collection tubes placed on ice and immediately stored at −80 °C. Additionally, detailed information on body weight and height was collected during the clinical visit by trained staff using standardized protocols to calculate BMI (kg/m^2^). Offspring BMI was transformed to age- and sex-specific standard deviation scores (BMI-SDS) according to German reference data [27].

The present analysis was restricted to one mother–offspring pair per family. Of 129 eligible mother–offspring pairs, 11 were excluded, because mothers were diagnosed with type 2 diabetes before or at the clinical study visit and type 2 diabetes has been associated with changes in the metabolomic profile, including sugars, branched-chain amino acids (BCAAs), intermediates of BCAA metabolism, and free fatty acids [28]. None of the offspring were diagnosed with type 2 diabetes at the time of the visit. From the remaining 118 mother–offspring pairs, 70 pairs had to be excluded due to incomplete or missing oral glucose tolerance tests for the offspring. The final sample consisted of 288 plasma samples from 48 mother–offspring pairs with complete metabolomics data (Appendix E).

All participants provided written informed consent to participate in the study. The study was approved by the Ethical Committee of the Technische Universität München, Munich, Germany (no. 2937).

### 4.2. Measurement of Insulin and Plasma Glucose

Plasma insulin was analyzed by immunoassay using the AIA-360 Analyzer (Tosoh Bioscience, Tokyo, Japan). Plasma glucose was measured routinely (Medizet, Munich, Germany) using a photometer (ARCHITECT c16000, Abbott, Abbott Park, IL, USA). Matsuda insulin sensitivity index (ISI-M) was applied to estimate insulin sensitivity, as it has been reported to be strongly correlated with the gold standard method using the hyperinsulinemic–euglycemic insulin clamp technique in children and adolescents [29,30]. ISI-M was calculated as follows: ISI-M = 10,000/square root of [(mean plasma glucose × mean plasma insulin during OGTT) × (fasting plasma glucose × fasting plasma insulin)] [31].

### 4.3. Dietary Behavior

Dietary information of the mothers was assessed during the clinical study visit several years postpartum with a validated food frequency questionnaire (FFQ) reflecting their dietary habits with regard to 85 food items during the preceding 4 weeks [32]. Dietary information of the children was collected by 3-day dietary food records, as described previously [26]. The diet records were reviewed by trained study personnel for plausibility and entered into a food database (PRODI^®^ 5 basis, Wissenschaftliche Verlagsgesellschaft, Stuttgart, Germany) to calculate daily intake of energy, nutrients, and food groups. For this study, intake of macronutrients was expressed as % of total energy intake. A glycemic index (GI) value was assigned to each carbohydrate-containing food in the FFQ or 3-day dietary records using the Diogenes reference database for Germany [33]. Foods with a carbohydrate (CHO) content below 1 g/100 g were not included in the calculation. The glycemic load of each individual diet was calculated by the sum of the CHO content (g) of each food item multiplied by the daily consumed amount of this item and the food’s GI (%), divided by 100. Additionally, a modified Dietary Approach to Stop Hypertension (DASH) score was calculated based on daily consumption of the following food groups: whole grains, fruits, vegetables, nuts/legumes, low-fat dairy products (≤1.5% fat content), red or processed meat, sweet snacks, salty snacks, sweetened beverages, and sodium. Because the daily energy intake differed across age groups, we calculated the energy-specific intake of each food group (intake per 1000 kcal). The modified DASH score was calculated according to [34]. Briefly, for each food group, mothers and offspring were separately classified into quintiles according to their daily intake/1000 kcal. For “healthy” food groups (fruits, vegetables, nuts/legumes, low-fat dairy products, and whole grains), quintile 1 (lowest daily intake) was assigned 1 point and quintile 5 was assigned 5 points. For “unhealthy” food groups (red or processed meat, sweet snacks, salty snacks, sweetened beverages, sodium), quintile 1 was assigned 5 points and quintile 5 was assigned 1 point. The DASH score was calculated as the sum of scores for each individual food group.

### 4.4. Physical Activity

Measures of overall physical activity (metabolic equivalent of task (MET) hours per week) were assessed with validated questionnaires. Maternal physical activity was assessed by the Freiburger Fragebogen zur körperlichen Aktivität [35], and offspring physical activity by the Motorik-Modul Aktivitätsfragebogen (MoMo-AFB) [36]. The overall activity index was determined by summing the MET-h per week for everyday basic and sporting activities [37]. For mothers, basic activities (walking, bicycling, gardening, and stair climbing), leisure activities (bike tours, swimming, dancing, and bowling), and other sports were included in the overall activity index. For the children, activities included supervised activities in preschool or school (sports lessons and extracurricular activities), club sports, leisure sports, and everyday activities such as walking, bicycling, and playing outside.

### 4.5. Metabolomics Analysis

Metabolomic measurements were performed at the Genome Analysis Centre of the Helmholtz Zentrum München, Germany. Plasma metabolites were measured with electrospray ionization–flow injection–tandem mass spectrometry (ESI-FIA-MS/MS) and electrospray ionization–liquid chromatography–tandem mass spectrometry (ESI-LC-MS/MS) with the Absolute*IDQ*™ p180 Kit (Biocrates Life Sciences AG, Innsbruck, Austria). This assay allows the simultaneous quantification of 188 metabolites in 10 µL of plasma. The assay procedures of the Absolute*IDQ*^TM^ p180 Kit and the metabolite nomenclature have been described in detail previously [38]. Briefly, 10 μL of plasma was pipetted onto a filter that was incorporated in a 96-well sandwich plate that already contained stable isotope-labeled internal standards. Amino acids were derivatized with 5% phenyl isothiocyanate reagent. The metabolites and internal standards were extracted with 5 mM ammonium acetate in methanol and the solution was centrifuged through a filter membrane. One part of the solution was diluted with running solvent for the FIA-MS/MS measurements, and another part was diluted with water for LC-MS/MS.

Quantification of metabolite concentrations and quality assessment were performed with Analyst 1.5 and 1.6 and Met*IDQ*™ software, which is an integral part of the Absolute*IDQ*™ p180 Kit. The concentration of each metabolite was calculated with reference to the appropriate internal standard set by the manufacturer and was reported in μmol/L. Fifty-one of 188 metabolites were excluded from further analysis if at least one of the following conditions was present: a coefficient of variation greater than 25% in 32 aliquots of a reference plasma sample, which were measured in parallel with the study samples for quality control; metabolites, for which the measurements were zero in more than 95% of the samples; metabolites, for which the measurements were below the limit of detection (= mean [21 blanks] + 3SD [21 blanks]) in more than 95% of the samples. For all other metabolites, measurements which were below the limit of detection were kept as values calculated in Met*IDQ*™ in the analysis instead of imputation, as GGMs have been reported to be very robust against application of different methods concerning data handling of values below the limit of detection [39]. In total, our data set included 13 metabolites for which the concentration in at least one sample was below the LOD. These included, in particular, acylcarnitines with medium-chain fatty acids and their derivatives with expected low frequencies (e.g., AC C10:1, AC C12, AC C12:1, AC C14:1-OH).

### 4.6. Statistical Analysis

To test whether the included mother–offspring pairs were different in demographic or clinical characteristics from pairs who were excluded due to missing OGTTs, Mann–Whitney U test (for continuous variables) or chi-square test (for categorical variables) was applied.

Correlations between BMI/BMI-SDS, ISI-M, physical activity (MET-h/week), and dietary variables within and between generations were assessed by Spearman’s correlations.

Metabolite concentrations of mothers and their offspring were quotient normalized and log-transformed, as has been recommended for normalizing omics data [14,40]. To depict the metabolic response to glucose challenge, the area under the curve (AUC) was calculated for each maternal and offspring metabolite from metabolite concentrations obtained at 0, 30, and 120 min during the OGTT. Intergenerational correlations of same metabolites were assessed by Gaussian graphical models (GGMs) using the GeneNet package [41]. GGMs were calculated based on the full-order partial Pearson correlation coefficients (i.e., pairwise correlations of metabolite AUCs within and across generations corrected for all remaining metabolite AUCs). GGMs were additionally corrected for maternal and offspring BMI/BMI-SDS, ISI-M, and age at the study visit and offspring sex by including the variables in the partial correlation calculation. Metabolites were considered to be significantly correlated between mothers and offspring after correction for multiple testing by applying the Benjamini–Hochberg approach for adjusting the *p*-values by controlling the false discovery rate (FDR) at 5%. Identified networks, including correlations between AUCs of metabolites within generations and AUCs of same metabolites between generations, were exported to Cytoscape for visualization [42]. The minimum partial correlation between generations, which was significant at FDR > 0.05, was selected as cut-off for the visualization of intragenerational correlations. In a sensitivity analysis, similar GGMs were calculated from metabolite concentrations obtained at 30 min and 120 min during OGTT.

To investigate whether postpartum dietary or physical activity behaviors affect AUCs of intergenerationally correlated metabolites in mothers and offspring, Spearman’s correlation coefficients were calculated for intergenerationally correlated metabolites and dietary glycemic load and MET-h/week separately in mothers and offspring. For the comparison of the AUCs of metabolites between mothers with or without impaired fasting glucose or impaired glucose tolerance, the Mann–Whitney U test was applied. Statistical analyses were performed with R software version 3.4.0.

## Figures and Tables

**Table 1:** Characteristics of the study cohort.

	Mothers		Offspring	
	N		N	
Female n (%)	48	48 (100)	48	23 (48)
Age at postpartum follow-up (years), median (IQR)	48	42.1 (38.0; 44.2)	48	8.0 (5.4; 8.8)
BMI (kg/m^2^, mothers), BMI-SDS (offspring), median (IQR)	48	26.4 (22.6; 32.0)	48	0.1 (−0.6; 0.8)
Overweight * n (%)	48	28 (58)	48	7 (15)
Glycemic load, median (IQR)	47	145.1 (114.8; 179.3)	31	100.3 (84.4; 131.1)
Physical activity (MET-h/week), median (IQR)	37	29.0 (18.0; 43.4)	20	71.0 (45.1; 103.6)
ISI-Matsuda median (IQR)	48	5.9 (3.6; 9.0)	43	10.3 (6.5; 13.8)

IQR, interquartile range; BMI, body mass index; SDS, standard deviation score; ISI, insulin sensitivity index; MET-h, metabolic equivalent of task hours. * Overweight: defined as BMI ≥ 25 kg/m^2^ in mothers and BMI-SDS > 1 in offspring.

**Table 2:** Intragenerational correlation of BMI/BMI-SDS, ISI-M, dietary glycemic load, and physical activity (MET-h/week) in mothers with gestational diabetes mellitus (GDM) and their offspring assessed at a median of 8 years after delivery. Results are presented as Spearman’s correlation coefficients (r). * Significant at *p* < 0.05.

	Mothers				Offspring			
Variable	BMI	ISI-M	Glycemic Load	MET-h/Week	BMI-SDS	ISI-M	Glycemic Load	MET-h/Week
BMI/BMI-SDS	1	−0.69 *	0.16	−0.10	1	−0.32*	0.21	−0.05
ISI-M	−0.69 *	1	−0.22	0.05	−0.32 *	1	−0.03	−0.07
Glycemic load	0.16	−0.22	1	0.2	0.21	−0.03	1	0.51 *
MET-h/week	−0.10	0.05	0.2	1	−0.05	−0.07	0.51 *	1

**Table 3:** Intragenerational correlations of AUC of metabolites with dietary glycemic load and physical activity (MET-h/week) in mothers with GDM and their offspring at a median of 8 years after delivery. * Results are presented as Spearman’s correlation coefficients.

Metabolite (AUC)	Dietary Glycemic Load				Physical Activity (MET-h/Week)			
	Mothers		Offspring		Mothers		Offspring	
	r *	*p*-Value	r *	*p*-Value	r *	*p*-Value	r *	*p*-Value
Carnitine (C0)	−0.02	0.9	0.28	0.12	0.06	0.72	0.20	0.40
Taurine	0.01	0.93	−0.21	0.25	0.07	0.81	0.30	0.20
Proline	−0.06	0.69	0.07	0.72	0.04	0.81	0.30	0.20
SM –(OH) C14:1	0.26	0.08	−0.15	0.42	0.32	0.06	−0.27	0.26
Creatinine	0.19	0.20	0.05	0.77	0.50	<0.01	0.10	0.84
PC ae C34:3	−0.01	0.51	0.001	1.0	0.16	0.35	0.15	0.52

**Figure A1:**
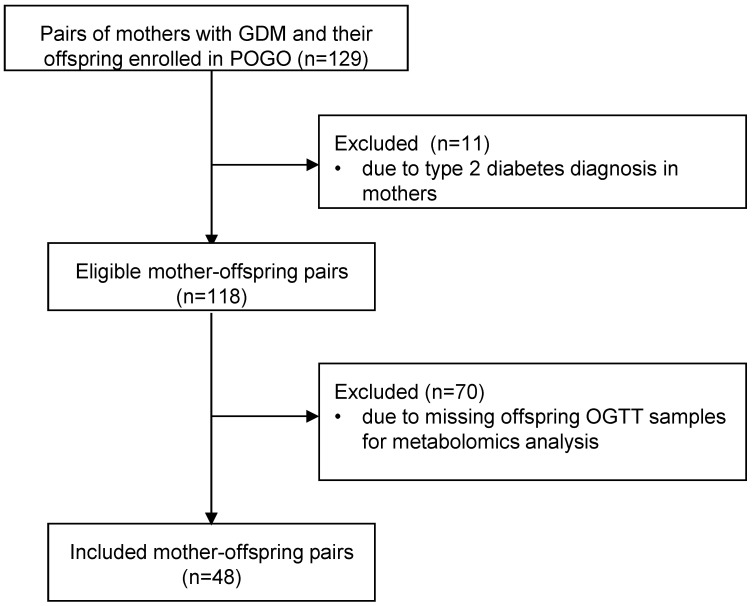
Flow-chart of mother-offspring pairs included in the study.

## Abbreviations

GDM	Gestational diabetes mellitus
OGTT	Oral glucose tolerance test
SDS	Standard deviation score
ISI-M	Matsuda insulin sensitivity index
FFQ	Food frequency questionnaire
GI	Glycemic index
CHO	Carbohydrate
MET	Metabolic equivalent of task
GGM	Gaussian graphical model
FDR	False discovery rate
PC	Phosphatidylcholine
AC	Acylcarnitine
SM	Sphingomyelin
Lyso-PC	Acyl-lysophosphatidylcholine
PCaa	Diacyl-phosphatidylcholine
PCae	Acyl-alkyl-phosphatidylcholine
DASH	Dietary Approach to Stop Hypertension
AUC	Area under the curve

## Appendix A

**Table A1:** Characteristics of mother–offspring pairs included in the study and mother–offspring pairs excluded due to missing oral glucose tolerance tests for the offspring.

	Included Mother–Offspring Pairs		Excluded Mother–Offspring Pairs		
	N		N		*p*-Value
Country of origin Germany, n (%)	48	40 (83.3%)	70	57 (81.4%)	0.40
Offspring sex, female n (%)	48	23 (48%)	70	31 (44%)	0.7
Maternal age at follow-up (years), median (IQR)	48	42.1 (38.0; 44.2)	70	40.2 (35.2; 43.5)	0.1
Offspring age at follow-up (years), median (IQR)	48	8.0 (5.4; 8.8)	70	5.1 (4.1; 7.4)	<0.0001
Maternal BMI at follow-up (kg/m^2^), median (IQR)	48	26.4 (22.6; 32.0)	70	25.3 (22.5; 30.4)	0.5
Offspring BMI-SDS, median (IQR)	48	0.1 (−0.6; 0.8)	47	0.04 (−0.8; 0.5)	0.3
Maternal fasting glucose (mg/dl), median (IQR)	48	94 (87; 103)	70	91 (87; 100)	0.2
Offspring fasting glucose (mg/dl), median (IQR)	48	88 (84; 92)	34	84 (80; 92)	0.06

## Appendix B

**Table A2:** Intergenerational correlations of daily intake of energy, protein, carbohydrates, fiber, fat, fatty acids, and DASH score in mothers with GDM and their offspring at a median of 8 years after delivery. Results are presented as Spearman’s correlation coefficients.

	Intergenerational Correlation	
	Correlation Estimate r	*p*-Value
Energy and Nutrients		
Energy (kcal/day)	−0.05	0.83
Protein (% of total EI)	0.17	0.47
Carbohydrates (% of total EI)	0.21	0.37
Fiber (g/day)	−0.10	0.60
Total Fat (% of total EI)	−0.06	0.78
Polyunsaturated fatty acids (% of total EI)	0.40	0.07
Monounsaturated fatty acids (% of total EI)	0.17	0.46
Saturated fatty acids (% of total EI)	0.08	0.74
DASH score	0.24	0.29

DASH, Dietary Approaches to Stop Hypertension; modified according to [34], calculated based on daily intake of the following food groups: whole grains, fruits, vegetables, nuts/legumes, low-fat dairy, red/processed meat, sweet snacks, salty snacks, sweetened beverages, sodium. EI, energy intake.

## Appendix C

**Table A3:** Correlations of AUC of plasma metabolite levels between mothers with GDM and their offspring and within generations at a median of 8 years after delivery (adjusted for maternal and offspring BMI/BMI-SDS, ISI-M, age, and offspring sex and all remaining metabolite AUCs, and significant at false discovery rate corrected *p*-value < 0.05). Results are shown for all metabolites with a partial correlation of >0.06 (minimum partial correlation for intergenerational correlations at FDR > 0.05) assessed by GGM, sorted according to inter- and intragenerational correlations and correlation strength.

Node1	Node2	Estimate r	P Raw	P FDR
Intergenerational correlations of same metabolites between mothers and offspring				
Carnitine		0.070	<0.00001	<0.01
Taurine		0.069	<0.00001	<0.01
Proline		0.065	<0.00001	<0.01
SM –OH C14:1		0.061	0.0001	0.01
Creatinine		0.061	0.0001	0.01
PC ae C34:3		0.060	0.0001	<0.05
Offspring correlations				
Lyso PC a C16:0	Lyso PC a C18:0	0.118	<0.00001	<0.0001
Leucine	Valine	0.115	<0.00001	<0.0001
AC C5	Kynurenine	0.112	<0.00001	<0.0001
PC aa C40:4	PC aa C40:5	0.111	<0.00001	<0.0001
Isoleucine	Leucine	0.110	<0.00001	<0.0001
AC C4:1	AC C6:1	0.110	<0.00001	<0.0001
SM C18:0	SM C18:1	0.107	<0.00001	<0.0001
PC ae C34:2	PC ae C36:3	0.106	<0.00001	<0.0001
PC aa C36:4	PC aa C38:4	0.104	<0.00001	<0.0001
Citrulline	Taurine	0.103	<0.00001	<0.0001
SM (-OH) C22:1	SM (-OH) C24:1	0.103	<0.00001	<0.0001
PC ae C42:4	PC ae C44:4	0.101	<0.00001	<0.0001
PC ae C32:1	PC ae C32:2	0.101	<0.00001	<0.0001
PC aa C34:2	PC aa C36:2	0.101	<0.00001	<0.0001
SM (-OH) C14:1c	SM (-OH) C16:1	0.100	<0.00001	<0.0001
SM -C24:0	SM (-OH) C22:1	0.094	<0.00001	<0.0001
PC ae C44:5	PC ae C44:6	0.093	<0.00001	<0.0001
PC aa C38:6	PC aa C40:6	0.092	<0.00001	<0.0001
PC aa C34:1	PC aa C36:1	0.091	<0.00001	<0.0001
AC C6:1	Valine	0.090	<0.00001	<0.0001
Kynurenine	Phenylalanine	0.089	<0.00001	<0.0001
Histidine	Serine	0.088	<0.00001	<0.0001
PC ae C42:5	PC ae C44:5	0.088	<0.00001	<0.0001
PC ae C40:4	PC ae C42:4	0.088	<0.00001	<0.0001
Isoleucine	Valine	0.087	<0.00001	<0.0001
SM C24:0	SM (-OH) C24:1	0.086	<0.00001	<0.0001
Carnitine	AC C3	0.086	<0.00001	<0.0001
SM (-OH) C22:1	SM (-OH) C22:2	0.086	<0.00001	<0.0001
LysoPC a C18:2	LysoPC a C20:4	0.085	<0.00001	<0.0001
SM C18:0	SM (-OH) C16:1	0.085	<0.00001	<0.0001
PC ae C38:4	PC ae C40:4	0.084	<0.00001	<0.0001
PC aa C42:5	PC aa C42:6	0.083	<0.00001	<0.0001
Serine	Threonine	0.083	<0.00001	<0.0001
AC C16	AC C18	0.082	<0.00001	<0.0001
PC ae C34:3	PC ae C36:5	0.082	<0.00001	<0.0001
PC aa C40:5	PC aa C42:5	0.082	<0.00001	<0.0001
PC aa C38:5	PC aa C40:5	0.081	<0.00001	<0.0001
PC ae C36:4	PC ae C38:5	0.081	<0.00001	<0.0001
PC ae C44:4	PC ae C44:5	0.081	<0.00001	<0.0001
AC C18:1	AC C18:2	0.081	<0.00001	<0.0001
LysoPC a C20:3	LysoPC a C20:4	0.081	<0.00001	<0.001
AC C4-OH	AC C4:1	0.080	<0.00001	<0.001
AC C2	AC C4-OH	0.080	<0.00001	<0.001
LysoPC a C18:1	LysoPC a C18:2c	0.079	<0.00001	<0.001
PC aa C38:6	PC ae C38:0	0.078	<0.00001	<0.001
PC ae C42:5	PC ae C44:6	0.078	<0.00001	<0.001
LysoPC a C18:2	LysoPC a C20:3	0.078	<0.00001	<0.001
Glycine	Phenylalanine	0.078	<0.00001	<0.001
LysoPC a C18:1	LysoPC a C20:3	0.078	<0.00001	<0.001
PC ae C38:4	PC ae C40:5	0.077	<0.00001	<0.001
SM C26:1	SM (-OH) C24:1	0.077	<0.00001	<0.001
PC aa C38:0	PC ae C38:6	0.077	<0.00001	<0.001
PC aa C30:0	PC ae C30:0	0.077	<0.00001	<0.001
PC aa C30:0	PC aa C32:0	0.077	<0.00001	<0.001
PC ae C40:4	PC ae C40:5	0.077	<0.00001	<0.001
PC ae C42:4	PC ae C44:5	0.077	<0.00001	<0.001
AC C10:2	Creatinine	0.076	<0.00001	<0.001
Glutamine	Lysine	0.076	<0.00001	<0.001
PC aaC40:4	PC aaC42:5	0.076	<0.00001	<0.001
Citrulline	Glycine	0.076	<0.00001	<0.001
AC C18	Taurine	0.076	<0.00001	<0.001
PC ae C42:4	PC ae C42:5	0.076	<0.00001	<0.001
LysoPC a C16:0	LysoPC a C17:0	0.075	<0.00001	<0.001
PC aa C42:0	PC aa C42:1	0.075	<0.00001	<0.001
PC ae C40:4	PC ae C42:5	0.075	<0.00001	<0.001
Asparagine	Serine	0.074	<0.00001	<0.001
SM C16:1	SM C18:1	0.074	<0.00001	<0.001
PC aa C38:4	PC aa C40:4	0.073	<0.00001	<0.001
LysoPC a C18:1	LysoPC a C20:4	0.073	<0.00001	0.001
PC ae C36:5	PC ae C38:6	0.073	<0.00001	0.001
AC C16:1	AC C4:1	0.073	<0.00001	0.001
PC aa C42:0	PC ae C44:6	0.073	<0.00001	0.001
LysoPC a C18:0	LysoPC a C20:4	0.073	<0.00001	0.001
PC aa C32:2	PC aa C34:4	0.073	<0.00001	<0.01
LysoPC a C17:0	LysoPC a C18:0	0.072	<0.00001	<0.01
PC aa C38:6	PC ae C40:6	0.072	<0.00001	<0.01
PC aa C40:4	PC aa C42:6	0.072	<0.00001	<0.01
AC C12	AC C14	0.071	<0.00001	<0.01
AC C16:1	AC C4-OH (C3DC)	0.071	<0.00001	<0.01
PC aa C40:6	PC ae C40:6	0.071	<0.00001	<0.01
PC aa C38:0	PC ae C40:6	0.071	<0.00001	<0.01
SM C16:0	SM C16:1	0.070	<0.00001	<0.01
PC ae C40:5	PC ae C40:6	0.070	<0.00001	<0.01
PC aa C36:2	PC ae C34:3	0.070	<0.00001	<0.01
PC aa C40:3	PC aa C42:5	0.070	<0.00001	<0.01
PC aa C36:6	PC ae C38:0	0.070	<0.00001	<0.01
SM C16:0	SM C18:0	0.070	<0.00001	<0.01
PC aa C40:2	PC aa C40:3	0.070	<0.00001	<0.01
PC aa C38:5	PC aa C40:4	0.069	<0.00001	<0.01
LysoPC a C16:0	LysoPC a C16:1	0.069	<0.00001	<0.01
LysoPC a C16:0	LysoPC a C20:4	0.069	<0.00001	<0.01
AC C10:2	Kynurenine	0.068	<0.00001	<0.01
PC ae C38:4	PC ae C38:5	0.068	<0.00001	<0.01
PC ae C42:5	PC ae C44:4	0.067	<0.00001	<0.01
Glutamine	Serine	0.067	<0.00001	<0.01
PC aa C36:3	PC aa C38:3	0.067	<0.00001	<0.01
AC C3	AC C4	0.067	<0.00001	<0.01
PC aa C40:2	PC aa C42:4	0.067	<0.00001	<0.01
PC aa C40:2	PC ae C42:1	0.067	<0.00001	<0.01
AC C14	AC C16	0.066	<0.00001	<0.01
PC aa C38:3	PC aa C40:4	0.066	<0.00001	<0.01
PC aa C32:0	PC ae C32:1	0.066	<0.00001	<0.01
PC aa C38:4	PC aa C40:5	0.066	<0.00001	<0.01
Arginine	Methionine	0.066	<0.00001	<0.01
SM C16:0	SM C24:1	0.065	<0.00001	<0.01
AC C16	AC C18:1	0.065	<0.00001	<0.01
LysoPC a C16:0	LysoPC a C18:1	0.065	<0.00001	<0.01
Leucine	Serine	0.065	<0.00001	<0.01
PC aa C38:4	PC aa C38:5	0.065	<0.00001	<0.01
PC ae C42:1	PC ae C42:2	0.064	<0.00001	<0.01
Glutamine	Histidine	0.064	<0.00001	<0.01
PC ae C36:4	PC ae C36:5	0.064	0.0001	<0.01
Histidine	Lysine	0.064	0.0001	<0.01
PC aa C42:4	PC aa C42:5	0.064	0.0001	<0.01
PC aa C40:3	PC aa C42:4	0.064	0.0001	<0.01
PC aa C34:2	PC aa C36:4	0.064	0.0001	<0.01
PC ae C40:2	SM (-OH) C24:1	0.064	0.0001	<0.01
Methionine	Tryptophan	0.064	0.0001	<0.01
PC aa C38:3	PC aa C40:5	0.064	0.0001	<0.01
PC aa C40:2	PC ae C42:2	0.064	0.0001	<0.01
LysoPC a C20:4	PC aa C36:4	0.063	0.0001	<0.01
PC aa C42:0	PC ae C42:5	0.063	0.0001	<0.01
LysoPC a C16:0	LysoPC a C20:3	0.063	0.0001	<0.01
Methionine	Phenylalanine	0.063	0.0001	<0.01
PC aa C32:1	PC aa C36:1	0.063	0.0001	<0.01
Lysine	Methionine	0.063	0.0001	<0.01
AC C14	AC C14:1-OH	0.063	0.0001	0.01
PC ae C36:4	PC ae C38:4	0.063	0.0001	0.01
PC aa C36:1	PC ae C34:1	0.062	0.0001	0.01
PC aa C36:4	PC aa C38:5	0.062	0.0001	0.01
SM C18:1	SM (OH) C16:1	0.062	0.0001	<0.05
PC ae C40:5	PC ae C42:5	0.062	0.0001	<0.05
AC C2	AC C4:1	0.062	0.0001	<0.05
LysoPC a C16:1	LysoPC a C18:1	0.062	0.0001	<0.05
PC aa C32:2	PC aa C36:6	0.062	0.0001	<0.05
PC aa C32:1	PC aa C34:1	0.062	0.0001	<0.05
PC ae C36:5	PC ae C38:5	0.062	0.0001	<0.05
PC ae C38:2	PC ae C40:3	0.062	0.0001	<0.05
PC aa C36:0	PC aa C38:0	0.061	0.0001	<0.05
AC C10	AC C12:1	0.061	0.0001	<0.05
PC aa C34:1	PC ae C36:0	0.061	0.0001	<0.05
Arginine	Glycine	0.061	0.0001	<0.05
PC ae C34:0	PC ae C34:1	0.061	0.0001	<0.05
Arginine	Glutamine	0.061	0.0001	<0.05
AC C18	AC C18:1	0.061	0.0001	<0.05
PC aa C38:1	PC aa C40:2	0.061	0.0001	<0.05
AC C12	AC C14:1	0.061	0.0001	<0.05
PC aa C36:4	PC ae C36:5	0.061	0.0001	<0.05
PC ae C38:6	PC ae C40:6	0.061	0.0001	<0.05
SM (OH) C16:1	SM (OH) C22:2	0.061	0.0001	<0.05
Phenylalanine	Proline	0.061	0.0001	<0.05
PC ae C36:4	PC ae C38:6	0.061	0.0001	<0.05
PC aa C40:2	PC aa C42:2	0.061	0.0001	<0.05
PC ae C32:1	PC ae C34:1	0.060	0.0001	<0.05
PC aa C30:0	PC aa C32:2	0.060	0.0001	<0.05
AC C4:1	Hexoses	0.060	0.0001	<0.05
PC aa C40:4	PC aa C42:4	0.060	0.0001	<0.05
AC C4	AC C6:1	0.060	0.0001	<0.05
PC aa C32:3	PC aa C34:4	0.060	0.0001	<0.05
AC C12	AC C12:1	0.060	0.0001	<0.05
PC aa C36:0	PC aa C36:1	−0.061	0.0001	<0.05
Carnitine	PC ae C36:0	−0.062	0.0001	<0.05
AC C4:1	Phenylalanine	−0.075	<0.00001	<0.001
Creatinine	SM (OH) C24:1	−0.078	<0.00001	<0.001
Maternal correlations				
PC aa C40:2	PC aa C40:3	0.112	<0.00001	<0.0001
Methionine	Threonine	0.111	<0.00001	<0.0001
PC ae C34:2	PC ae C36:3	0.107	<0.00001	<0.0001
PC aa C40:3	PC aa C42:5	0.099	<0.00001	<0.0001
Aspartate	Taurine	0.099	<0.00001	<0.0001
PC ae C42:4	PC ae C44:4	0.098	<0.00001	<0.0001
PC ae C42:5	PC ae C44:5	0.098	<0.00001	<0.0001
SM C16:1	SM C18:1	0.094	<0.00001	<0.0001
SM C18:1	SM C20:2	0.094	<0.00001	<0.0001
PC ae C36:4	PC ae C36:5	0.094	<0.00001	<0.0001
SM C24:0	SM (OH) C22:1	0.093	<0.00001	<0.0001
PC ae C36:4	PC ae C38:5	0.093	<0.00001	<0.0001
Alanine	Threonine	0.093	<0.00001	<0.0001
PC aa C40:4	PC aa C40:5	0.092	<0.00001	<0.0001
Carnitine	AC C2	0.092	<0.00001	<0.0001
SM C18:0	SM C18:1	0.091	<0.00001	<0.0001
Asparagine	Threonine	0.091	<0.00001	<0.0001
Isoleucine	Leucine	0.090	<0.00001	<0.0001
Leucine	Valine	0.089	<0.00001	<0.0001
PC ae C34:3	PC ae C36:5	0.089	<0.00001	<0.0001
PC aa C36:4	PC aa C38:4	0.089	<0.00001	<0.0001
SM (OH) C22:1	SM (OH) C24:1	0.088	<0.00001	<0.0001
PC ae C40:4	PC ae C42:4	0.088	<0.00001	<0.0001
SM C26:1	SM (OH) C24:1	0.088	<0.00001	<0.0001
Methionine	Tyrosine	0.088	<0.00001	<0.0001
PC ae C40:2	SM C26:1	0.087	<0.00001	<0.0001
AC C16	AC C18	0.086	<0.00001	<0.0001
SM (OH) C14:1	SM (OH) C16:1	0.086	<0.00001	<0.0001
PC aa C36:6	PC ae C38:0	0.086	<0.00001	<0.0001
PC ae C40:2	SM (OH) C24:1	0.085	<0.00001	<0.0001
Glycine	Serine	0.085	<0.00001	<0.0001
PC aa C38:6	PC aa C40:6	0.085	<0.00001	<0.0001
PC ae C44:4	PC ae C44:5	0.084	<0.00001	<0.0001
PC aa C36:5	PC aa C36:6	0.084	<0.00001	<0.0001
PC ae C36:4	PC ae C38:4	0.084	<0.00001	<0.0001
Citrulline	Threonine	0.083	<0.00001	<0.0001
Asparagine	Methionine	0.083	<0.00001	<0.0001
PC ae C36:2	PC ae C38:2	0.082	<0.00001	<0.0001
Isoleucine	Valine	0.082	<0.00001	0.0001
SM C16:1	SM C20:2	0.081	<0.00001	0.0001
AC C3	AC C4	0.081	<0.00001	0.0001
SM C24:0	SM C24:1	0.081	<0.00001	0.0001
PC ae C32:1	PC ae C32:2	0.080	<0.00001	<0.001
PC ae C38:4	PC ae C40:4	0.079	<0.00001	<0.001
AC C12	AC C2	0.079	<0.00001	<0.001
AC C4:1	AC C6:1	0.078	<0.00001	<0.001
LysoPC a C16:0	LysoPC a C18:0	0.077	<0.00001	<0.001
Citrulline	Methionine	0.076	<0.00001	<0.001
PC aa C32:2	PC aa C34:4	0.076	<0.00001	<0.001
SM C24:0	SM (OH) C24:1	0.075	<0.00001	<0.001
PC ae C34:2	PC ae C34:3	0.075	<0.00001	<0.001
Alanine	AC C10:2	0.075	<0.00001	<0.001
AC C16	AC C18:1	0.075	<0.00001	<0.001
PC ae C42:2	PC ae C42:3	0.075	<0.00001	<0.001
PC ae C34:3	PC ae C36:3	0.075	<0.00001	<0.001
PC aa C36:5	PC ae C38:0	0.075	<0.00001	<0.001
PC aa C34:4	PC aa C36:6c	0.074	<0.00001	<0.001
PC aa C38:5	PC aa C40:5	0.074	<0.00001	<0.001
PC ae C40:1	PC ae C42:1	0.074	<0.00001	<0.001
LysoPC a C18:1	LysoPC a C18:2	0.074	<0.00001	<0.001
AC C14	AC C16	0.073	<0.00001	<0.001
PC ae C42:4	PC ae C44:5	0.073	<0.00001	<0.001
LysoPC a C17:0	LysoPC a C18:0	0.073	<0.00001	<0.001
SM C20:2	SM (OH) C22:2	0.072	<0.00001	0.001
SM C24:1	SM C26:1	0.071	<0.00001	0.001
AC C10	AC C12:1	0.071	<0.00001	<0.01
PC aa C40:2	PC aa C42:5	0.071	<0.00001	<0.01
AC C10	AC C10:1	0.071	<0.00001	<0.01
LysoPC a C16:0	LysoPC a C17:0	0.071	<0.00001	<0.01
PC ae C36:3	PC ae C36:4	0.070	<0.00001	<0.01
PC ae C36:5	PC ae C38:6	0.070	<0.00001	<0.01
AC C18:1	AC C18:2	0.070	<0.00001	<0.01
PC aa C38:0	PC ae C38:6	0.070	<0.00001	<0.01
AC C14	AC C14:1-OH	0.069	<0.00001	<0.01
AC C10:1	AC C12:1	0.069	<0.00001	<0.01
PC aa C34:1	PC aa C36:1	0.069	<0.00001	<0.01
PC aa C36:0	PC ae C38:6	0.069	<0.00001	<0.01
PC ae C36:5	PC ae C38:5	0.069	<0.00001	<0.01
PC aa C40:2	PC aa C42:4	0.068	<0.00001	<0.01
PC aa C34:2	PC aa C36:2	0.068	<0.00001	<0.01
PC ae C40:5	PC ae C42:5	0.068	<0.00001	<0.01
LysoPC a C16:1	LysoPC a C20:3	0.068	<0.00001	<0.01
PC ae C34:2	PC ae C36:4	0.068	<0.00001	<0.01
PC ae C40:4	PC ae C44:5	0.068	<0.00001	<0.01
LysoPC a C16:0c	LysoPC a C18:1	0.068	<0.00001	<0.01
PC ae C40:4	PC ae C44:4	0.067	<0.00001	<0.01
Kynurenine	Tyrosine	0.067	<0.00001	<0.01
PC aa C42:0	PC aa C42:1	0.067	<0.00001	<0.01
PC ae C40:4	PC ae C42:5	0.066	<0.00001	<0.01
PC ae C42:1	PC ae C44:3	0.066	<0.00001	<0.01
AC C16	AC C2	0.066	<0.00001	<0.01
Asparagine	AC C10:2	0.066	<0.00001	<0.01
PC aa C42:0	PC ae C44:6	0.066	<0.00001	<0.01
Carnitine	Taurine	0.066	<0.00001	<0.01
Carnitine	AC C3	0.066	<0.00001	<0.01
PC aa C42:5	PC aa C42:6	0.066	<0.00001	<0.01
Aspartate	Ornithine	0.066	<0.00001	<0.01
Acetylornithine	Glycine	0.065	<0.00001	<0.01
PC aa C30:0	PC ae C30:0	0.065	<0.00001	<0.01
Acetylornithine	AC C4	0.065	<0.00001	<0.01
PC aa C32:3	PC aa C34:3	0.065	<0.00001	<0.01
Glutamine	Serine	0.065	<0.00001	<0.01
LysoPC a C18:1	LysoPC a C20:4	0.065	<0.00001	<0.01
PC aa C42:4	PC aa C42:5	0.065	<0.00001	<0.01
PC ae C42:4	PC ae C42:5	0.065	<0.00001	<0.01
AC C4:1	Hexoses	0.065	<0.00001	<0.01
Arginine	Histidine	0.064	<0.00001	<0.01
LysoPC a C16:0	LysoPC a C18:2	0.064	<0.00001	<0.01
PC aa C38:5	PC aa C42:6	0.064	0.0001	<0.01
LysoPC a C14:0	LysoPC a C16:1	0.064	0.0001	<0.01
PC aa C38:6	PC ae C38:0	0.064	0.0001	<0.01
Arginine	Threonine	0.064	0.0001	<0.01
AC C3	AC C6:1	0.064	0.0001	<0.01
PC ae C42:1	PC ae C42:2	0.064	0.0001	<0.01
AC C10	AC C12	0.064	0.0001	<0.01
Alanine	Asparagine	0.063	0.0001	<0.01
PC ae C40:3	PC ae C44:4	0.063	0.0001	<0.01
SM C16:1	SM (OH) C22:2	0.063	0.0001	<0.01
PC ae C36:4	PC ae C38:6	0.063	0.0001	<0.01
PC aa C42:0	PC ae C42:5	0.063	0.0001	0.01
LysoPC a C20:3	LysoPC a C20:4	0.062	0.0001	0.01
PC aa C30:0	PC ae C34:0	0.062	0.0001	0.01
AC C16:1	AC C6:1	0.062	0.0001	0.01
PC aa C38:4	PC aa C40:4	0.062	0.0001	0.01
SM C24:0	SM C26:1	0.062	0.0001	0.01
SM C20:2	SM C24:1	0.062	0.0001	0.01
AC C12	AC C14	0.061	0.0001	0.01
PC ae C42:5	PC ae C44:6	0.061	0.0001	0.01
LysoPC a C18:0	LysoPC a C18:2	0.061	0.0001	0.01
Isoleucine	Phenylalanine	0.061	0.0001	0.01
AC C14:1-OH	AC C7-DC	0.061	0.0001	0.01
AC C14:1-OH	AC C18	0.061	0.0001	0.01
AC C16:1	Phenylalanine	0.061	0.0001	0.01
PC aa C40:3	PC aa C42:4	0.061	0.0001	0.01
PC ae C30:0	PC ae C34:0	0.061	0.0001	0.01
AC C14	AC C14:1	0.061	0.0001	0.01
SM C16:0	SM C16:1	0.061	0.0001	0.01
Citrulline	Taurine	0.060	0.0001	<0.05
PC ae C38:5	PC ae C38:6	0.060	0.0001	<0.05
PC aa C36:5	PC aa C38:5	0.060	0.0001	<0.05
LysoPC a C18:0	LysoPC a C18:1	0.060	0.0001	<0.05
AC C18	PC aa C42:5	−0.060	0.0001	<0.05
Creatinine	PC ae C40:3	−0.062	0.0001	0.01
Carnitine	PC aa C32:0	−0.062	0.0001	0.01
Aspartate	PC aa C38:4	−0.062	0.0001	0.01
AC C18	SM C20:2	−0.067	0.0000	<0.01
Acetylornitine	SM C18:0	−0.068	0.0000	<0.01
Aspartate	PC ae C38:4	−0.070	0.0000	<0.01

PC aa, diacyl-phosphatidylcholine; PC ae: acyl-alkyl-phosphatidylcholine; LysoPC a, acyl-lysophatidyl-choline; SM, sphingomyelin; AC, acylcarnitine.

## Appendix D

**Table A4:** AUCs of six intergenerationally correlated metabolites (mean, SD) in mothers with GDM and impaired fasting glucose or impaired glucose tolerance and mothers with GDM and normal glucose tolerance at a median of 8 years after delivery.

Metabolites (AUC)	Mothers with Impaired Fasting Glucose or Impaired Glucose Tolerance		Mothers with Normal Glucose Tolerance		*p*-Value *
	Mean	SD	Mean	(SD)	
Carnitine (C0)	294.1	20.6	293.3	20.4	0.7
Creatinine	368.1	19.2	361.5	17.3	0.2
PC ae C34:3	95.9	28.1	105.9	27.5	0.2
	476.1	35.9	472.0	39.1	0.6
	116.3	22.3	103.4	19.4	0.05
	330.1	27.4	334.9	22.0	0.6

* Comparisons were performed by Mann–Whitney U test.
